# Searching for gene-gene interactions through variance quantitative trait loci of 29 continuous Taiwan Biobank phenotypes

**DOI:** 10.3389/fgene.2024.1357238

**Published:** 2024-03-07

**Authors:** Wan-Yu Lin

**Affiliations:** ^1^ Institute of Health Data Analytics and Statistics, College of Public Health, National Taiwan University, Taipei, Taiwan; ^2^ Master of Public Health Degree Program, College of Public Health, National Taiwan University, Taipei, Taiwan

**Keywords:** continuous trait, epistasis, homoscedasticity, heteroscedasticity, scale test

## Abstract

**Introduction:** After the era of genome-wide association studies (GWAS), thousands of genetic variants have been identified to exhibit main effects on human phenotypes. The next critical issue would be to explore the interplay between genes, the so-called “gene-gene interactions” (GxG) or epistasis. An exhaustive search for all single-nucleotide polymorphism (SNP) pairs is not recommended because this will induce a harsh penalty of multiple testing. Limiting the search of epistasis on SNPs reported by previous GWAS may miss essential interactions between SNPs without significant marginal effects. Moreover, most methods are computationally intensive and can be challenging to implement genome-wide.

**Methods:** I here searched for GxG through variance quantitative trait loci (vQTLs) of 29 continuous Taiwan Biobank (TWB) phenotypes. A discovery cohort of 86,536 and a replication cohort of 25,460 TWB individuals were analyzed, respectively.

**Results:** A total of 18 nearly independent vQTLs with linkage disequilibrium measure *r* ^*2*^ < 0.01 were identified and replicated from nine phenotypes. 15 significant GxG were found with *p*-values <1.1E-5 (in the discovery cohort) and false discovery rates <2% (in the replication cohort). Among these 15 GxG, 11 were detected for blood traits including red blood cells, hemoglobin, and hematocrit; 2 for total bilirubin; 1 for fasting glucose; and 1 for total cholesterol (TCHO). All GxG were observed for gene pairs on the same chromosome, except for the *APOA5* (chromosome 11)—*TOMM40* (chromosome 19) interaction for TCHO.

**Discussion:** This study provided a computationally feasible way to search for GxG genome-wide and applied this approach to 29 phenotypes.

## 1 Introduction

Over the past decade, thousands of genetic variants have been found to be responsible for disease risk (Uffelmann et al., 2021). The next critical topic is to explore “gene-gene interaction” (GxG), also known as “epistasis,” indicating that “the effect of a gene on a phenotype is dependent on another gene.” The importance of GxG has widely been recognized (Van Steen, 2012). However, due to the curse of multiplicity, GxG remains challenging to identify and replicate (Moore and Williams, 2009).

Some statistical methods have been proposed for identifying GxG (Ritchie et al., 2003; Lou et al., 2007). For example, by prioritizing single-nucleotide polymorphisms (SNPs) with prior biological knowledge, Ma et al. identified an interaction between the *LIPC* (on chromosome 15) and *HMGCR* (on chromosome 5) genes influencing high-density lipoprotein cholesterol (HDL-C) levels (Ma et al., 2015). To alleviate the penalty of multiple testing, Ma et al. only tested SNP pairs supported by prior knowledge, including the quantitative trait loci (QTLs) identified from genome-wide association studies (GWAS) of lipid traits.

The multifactor dimensionality reduction (MDR) approach is well-known for detecting GxG for binary disease outcomes (Ritchie et al., 2003). This method was later generalized for both binary and continuous phenotypes (Lou et al., 2007). However, the permutation testing to assess the statistical significance of GxG in MDR is computationally intensive (Pattin et al., 2009). It is impractical to analyze genome-wide pairwise SNPs through this MDR approach (Abo Alchamlat and Farnir, 2017). Although some computationally efficient methods were derived by extending MDR, they were mainly designed for case-control studies (Pattin et al., 2009; Abo Alchamlat and Farnir, 2017). It remains a challenging task to identify GxG from continuous traits on a genome-wide scale.

In addition to MDR, Machine learning (ML) approaches such as “random forests” (Botta et al., 2014) and “neural networks” (Motsinger et al., 2007) were also proposed to explore GxG. However, training the ML models is computationally expensive, and a genome-wide search for GxG is implausible. Therefore, investigators usually perform a variable selection before searching for GxG (Wu et al., 2018). Hence, critical SNPs can be missed during the variable selection process (Chattopadhyay and Lu, 2019).

The above-mentioned sophisticated models are difficult to implement on hundreds of thousands of SNPs. Another computationally feasible method is prioritizing SNPs via variance quantitative trait loci (vQTLs) (Marderstein et al., 2021; Westerman et al., 2022). Leveraging phenotypic variability across the three genotypes of an SNP can facilitate the discoveries of GxG or gene-environment interactions (GxE). In this study, I searched for GxG through vQTLs of 29 continuous Taiwan Biobank (TWB) phenotypes.

## 2 Materials and methods

### 2.1 Taiwan Biobank data

TWB was approved by the Institutional Review Board on Biomedical Science Research/IRB-BM, Academia Sinica, and the Ethics and Governance Council of Taiwan Biobank, Taiwan. TWB approved my application to access the data on 18 February 2020 (application number: TWBR10810-07). The current work further received approval from the Research Ethics Committee of the National Taiwan University Hospital (NTUH-REC no. 201805050RINB).

Since October 2012, TWB has recruited Taiwan residents aged 30 to 70 and collected their genomic and lifestyle factors (Chen et al., 2016). After signing informed consent, community-based volunteers took physical examinations and provided blood and urine samples. TWB health professionals collected lifestyle information through a face-to-face interview with each participant.

As of March 2022, 27,719 and 103,332 individuals (aged 30–70 years) have been whole-genome genotyped by the TWB 1.0 and TWB 2.0 genotyping arrays, respectively. The TWB 1.0 array was designed for Taiwan’s Han Chinese, running on the Axiom Genome-Wide Array Plate System (Affymetrix, Santa Clara, CA). The TWB 2.0 array was developed according to the experience of designing the TWB 1.0 array and the next-generation sequencing of ∼1,000 TWB individuals. These two arrays were released in April 2013 and August 2018, respectively. Because the sample size of “individuals genotyped by the TWB 2.0 array” (called “the TWB2 cohort”) was larger than that genotyped by the TWB 1.0 array (called “the TWB1 cohort”), the TWB2 cohort was treated as a discovery set. In contrast, the TWB1 cohort was used as a replication set.

A total of 1,462 individuals were genotyped by both arrays. To ensure that the replication set was independent of the discovery set, I removed these 1,462 individuals from the TWB2 cohort. I also tried to exclude individuals with more than 10% missing in their genotype calls, where 10% is a commonly adopted cutoff in quality control (Band et al., 2019). Nonetheless, no individuals were removed due to this criterion.

To remove cryptic relatedness, I calculated PI-HAT = Probability (IBD = 2) + 0.5 × Probability (IBD = 1) by PLINK 1.9 (Purcell et al., 2007), where IBD is the genome-wide identity by descent sharing coefficients between any two individuals. I excluded one individual from each pair with PI-HAT ≥ 0.2, a cutoff value chosen by some studies (Calabro et al., 2015; An et al., 2019; Lin et al., 2020a; Lin, 2022a; Lin, 2022b). After this step, the TWB2 (discovery) and TWB1 (replication) cohorts included 86,536 and 25,460 individuals, respectively.

TWB 2.0 and TWB 1.0 arrays comprised 648,611 and 632,172 autosomal SNPs, respectively. I excluded 17,419 SNPs with Hardy-Weinberg test *p*-values <5.7 × 10^−7^ (WTCCC, 2007) and 22,614 SNPs with genotyping rates <95% from the TWB2 cohort and removed 6,900 SNPs with Hardy-Weinberg test *p* values <5.7 × 10^−7^ (WTCCC, 2007) and 27,628 SNPs with genotyping rates <95% from the TWB1 cohort. The remaining 608,578 TWB2 SNPs and 597,644 TWB1 SNPs were used to construct ancestry principal components (PCs).

The Michigan Imputation Server (https://imputationserver.sph.umich.edu/index.html) was further used to impute genotypes. The East Asian population from the 1,000 Genomes Phase 3 v5 was chosen as the reference panel. I removed SNPs with low imputation information scores (R-square <0.8), with imputation rates <95%, or with Hardy-Weinberg test *p*-values <5.7 × 10^−7^ (WTCCC, 2007). The TWB2 and TWB1 individuals were finally genotyped (or imputed) on 6,546,183 and 7,433,014 autosomal SNPs, respectively.

With a larger sample size, TWB2 (*n* = 86,536) was treated as the discovery cohort. A total of 4,807,430 TWB2 SNPs with minor allele frequencies (MAFs) ≥ 5% were analyzed sequentially. I skipped the epistasis analysis for SNPs with MAFs <5% due to the inferior genotyping (or imputation) accuracy for low-frequency variants (Mitt et al., 2017). Moreover, GxG studies usually focus on common SNPs because of their better reproducibility (Wang et al., 2019). If the sample size of any genotype combination (from an SNP pair) is too small, this GxG evidence can hardly be replicated in another cohort. Significant SNPs would be further investigated using the TWB1 (*n* = 25,460) again, which was regarded as the replication cohort. Totally 29 traits in eight categories were investigated, including

(A) Six lung function traits: vital capacity, tidal volume, inspiratory reserve volume, expiratory reserve volume, forced vital capacity, and forced expiratory volume in 1 s.
(B) Four lipid traits: HDL, low-density lipoprotein cholesterol (LDL), total cholesterol (TCHO), and triglyceride (TG).
(C) Five obesity traits: BMI, body fat percentage (BFP), waist circumference (WC), hip circumference (HC), and waist-hip ratio (WHR).
(D) Five blood traits: red blood cells (RBC), white blood cells (WBC), platelets, hemoglobin (HB), and hematocrit (HCT).
(E) Three kidney traits: Creatinine, uric acid (UA), blood urea nitrogen.
(F) Two liver traits: total bilirubin (TB) and albumin.
(G) Two hypertension traits: diastolic and systolic blood pressure levels.
(H) Two diabetes traits: fasting glucose (FG) and glycated hemoglobin (HbA1c).

### 2.2 Variance quantitative trait loci (vQTLs)

The total number of pairwise interaction tests among 4,807,430 SNPs is very huge. Considering 29 phenotypes, conventionally, investigators need to perform $\left(\begin{array}{c} 4,807,430\\ 2 \end{array}\right)\times 29=3.35\times 10^{14}$ GxG tests. The power to detect GxG will be largely reduced due to the harsh penalty of multiple testing. In the following, I introduce a vQTL method to prioritize SNPs exhibiting GxG. Let *G*_*1*_ and *G*_*2*_ be the numbers of minor alleles at two SNPs. Without loss of generality, modeling a phenotype (denoted as “*Y*”) with the two SNPs (*G*_*1*_ and *G*_*2*_) can be expressed as follows,

$$Y=\beta_{0}+\beta_{G_{1}}G_{1}+\beta_{G_{2}}G_{2}+\beta_{\mathrm{INT}}G_{1}\times G_{2}+\varepsilon ,$$ (1)

where ε is the random error term. Conditional on the genotype of the first SNP, the variance of *Y* can be derived as

$$\begin{array}{ll} Var\left(\left.Y\right|G_{1}=g_{1}\right) & =Var\left(\beta_{0}+\beta_{G_{1}}G_{1}+\beta_{G_{2}}G_{2}+\beta_{\mathrm{INT}}G_{1}\times G_{2}+\left.\varepsilon \right|G_{1}=g_{1}\right)\\ & =Var\left(\beta_{0}\right)+Var\left(\beta_{G_{1}}G_{1}|G_{1}=g_{1}\right)+Var\left(\beta_{G_{2}}G_{2}\right)+Var\left(\beta_{\mathrm{INT}}G_{1}\times G_{2}|G_{1}=g_{1}\right)\\ & +2Cov\left(\beta_{G_{2}}G_{2},\beta_{\mathrm{INT}}G_{1}\times G_{2}|G_{1}=g_{1}\right)+Var\left(\varepsilon \right)=0+0+\beta_{G_{2}}^{2}Var\left(G_{2}\right)+\beta_{\mathrm{INT}}^{2}g_{1}^{2}Var\left(G_{2}\right)\\ & +2\beta_{G_{2}}\beta_{\mathrm{INT}}g_{1}Var\left(G_{2}\right)+Var\left(\varepsilon \right)={\left(\beta_{G_{2}}+\beta_{\mathrm{INT}}g_{1}\right)}^{2}Var\left(G_{2}\right)+Var\left(\varepsilon \right) \end{array}$$ (2)

I obtained $Var\left(\left.Y\right|G_{1}=g_{1}\right)={\left(\beta_{G_{2}}+\beta_{\mathrm{INT}}g_{1}\right)}^{2}Var\left(G_{2}\right)+Var\left(\varepsilon \right)$ . In the absence of GxG, $\beta_{\mathrm{INT}}=0$ and $Var\left(\left.Y\right|G_{1}=g_{1}\right)=\beta_{G_{2}}^{2}Var\left(G_{2}\right)+Var\left(\varepsilon \right)$ , representing the variance of *Y* remains constant across the three genotypes of the first SNP ( $g_{1}$ = 0, 1, and 2). Therefore, I investigated GxG by testing equal variance (homoscedasticity) of a phenotype across the three genotype groups of each SNP.

### 2.3 Genome-wide vQTL search for 29 TWB phenotypes

To provide results robust to outliers and the distributions of traits, I performed the “rank-based inverse normal transformation” (RINT) transformation (McCaw et al., 2020) on each trait before the vQTL analysis. RINT-trait was transformed to be normally distributed through this step. Subsequently, I obtained “genotypes-and-covariates adjusted RINT-trait” via the residuals of regressing RINT-trait on genotypes and covariates. Specifically, for each SNP, I adjusted RINT-trait with genotype effects as two dummy variables and covariates, including sex (male vs. female), age (in years), body mass index (BMI, in kg/m^2^), performing regular exercise (yes vs. no), educational attainment (an integer from 1 to 7), smoking status (yes vs. no), drinking status (yes vs. no), and the first 10 ancestry PCs.

The abovementioned covariates are commonly adjusted for continuous phenotypes, because each can influence the phenotypes to some extent (Lin et al., 2020b; Lin, 2022c). Current smoking indicated “having smoked cigarettes for at least 6 months and having not quit smoking when joining the TWB. Drinking was defined as “having a weekly intake of more than 150 mL of alcoholic beverages for at least 6 months and having not stopped drinking when joining the TWB.” Regular exercise was defined as “performing exercise lasting for 30 min thrice a week.” Educational attainment was an integer ranging from 1 to 7: 1 (illiterate), 2 (no formal education but literate), 3 (primary school graduate), 4 (junior high school graduate), 5 (senior high school graduate), 6 (college graduate), and 7 (Master’s or higher degree). When analyzing the five obesity traits (BMI, BFP, WC, HC, and WHR), BMI was excluded from the covariates.

Through the above step, I obtained the “genotypes-and-covariates adjusted RINT-trait,” denoted as “ $e_{i}$ ” for the *i*th individual (*i* = 1, 2, … , *n*). The dispersion of $e_{i}$ was then calculated by ${D_{i}=\left(e_{i}-\tilde{e}\right)}^{2}$ , where $\tilde{e}$ was the sample median of $e_{i}$ across all *n* individuals. Because of the robustness, the sample median is adopted instead of the sample mean. Subsequently, I regressed $D_{i}$ on the two dummy variables for genotype coding to check whether the dispersion of $e_{i}$ was dependent on different genotypes. The significance of the *F*-statistic of this regression model meant that the dispersion of “genotypes-and-covariates adjusted RINT-trait” ( $e_{i}$ ) varied with different genotypes, which was a clue of GxG according to the derivation of Equation 2. This is called the “scale test” for detecting trait heteroscedasticity across genotypes.

This scale test for vQTL identification is not novel. Several studies have previously introduced it (Pare et al., 2010; Soave et al., 2015; Soave and Sun, 2017; Staley et al., 2022; Westerman et al., 2022). Some investigators used Levene’s statistics (Levene, 1960) to test for equal variance (Pare et al., 2010; Westerman et al., 2022), while others reformulated the concept to a regression framework (Soave et al., 2015; Soave and Sun, 2017; Staley et al., 2022). However, these two approaches are conceptually identical. The workflow shown here is based on the regression framework with the potential to allow for continuous exposures (Soave et al., 2015; Soave and Sun, 2017; Staley et al., 2022). The R code to implement this regression for vQTL testing can be downloaded from https://github.com/WanYuLin/Univariate-scale-test-UST.

I analyzed 4,807,430 SNPs with MAFs ≥ 5% in TWB2. *p*-values of the scale test <0.05/(4,807,430 × 29) = 3.6E-10 were considered significant. Significant SNPs identified from the TWB2 cohort were further analyzed using the TWB1 cohort. SNPs with *p*-values of the scale test <0.05/(the number of SNPs analyzed in TWB1) were considered successfully replicated. These SNPs were called the vQTLs.

Based on the replicated vQTLs, I then performed an over-representation analysis on the Reactome pathway webpage (Fabregat et al., 2017) (https://reactome.org/). Over-representation analysis assesses whether some Reactome pathways are enriched (over-represented) in the vQTLs identified within the same trait category. It answers the question, “Do the vQTLs from a trait category contain more proteins for a certain pathway than that is expected by chance?” This over-representation test generates a *p*-value based on the hypergeometric distribution, which is then corrected by the Benjamini–Hochberg false discovery rate (FDR) procedure (Benjamini and Hochberg, 1995) (https://reactome.org/userguide/analysis).

### 2.4 Simulation studies

Although the scale test is not novel, I still conducted a simulation study to assess its power of detecting GxG, given the sample sizes of TWB2 and TWB1. Without loss of generality, four SNPs (rs150856817 on chromosome 2, rs2075291 and rs662799 on chromosome 11, and rs4144003 on chromosome 16) were, in turn (one by one), regarded as SNP2. The TWB2 and TWB1 SNPs on chromosome 1 were, in turn, treated as SNP1. As derived by Equation 2, $Var\left(\left.Y\right|G_{1}=g_{1}\right)={\left(\beta_{G_{2}}+\beta_{\mathrm{INT}}g_{1}\right)}^{2}Var\left(G_{2}\right)+Var\left(\varepsilon \right)$ . I tested whether the phenotypic variance depended on the three genotypes of SNP1. The trait value was then generated as follows,

$$Y_{i}=\beta_{G_{1}}G_{1i}+\beta_{G_{2}}G_{2i}+\beta_{\mathrm{INT}}G_{1i}\times G_{2i}+\varepsilon_{i},i=1,\cdots ,86536\;\left(\text{or }25460\right),$$ (3)

where $G_{1i},G_{2i}=0$ , 1, 2 represented the numbers of minor alleles at SNP1 and SNP2, respectively. The random error term $\varepsilon_{i}$ was generated from a standard normal distribution with a mean of 0 and a variance of 1. I considered two situations, as follows,

(I) **Without SNP main effects:** By specifying $\beta_{G_{1}}=\beta_{G_{2}}=\beta_{\mathrm{INT}}=0$ (scenario 1), I evaluated the type I error rates given the significance level of 3.6E-10. To demonstrate that this method is also capable of detecting “pure epistasis” (no individual SNP main effects) (Russ et al., 2022), power was assessed at $\beta_{G_{1}}=\beta_{G_{2}}=0$ and $\beta_{\mathrm{INT}}=0.3$ . Because the four SNPs were, in turn, regarded as SNP2, the power was evaluated under scenarios 2–5. A total of 10^8^ replications were conducted under this situation, and each scenario was investigated with $2\times 10^{7}$ simulation replicates.
(II) **With SNP main effects:** By specifying $\beta_{G_{1}}=\beta_{G_{2}}=0.3$ and $\beta_{\mathrm{INT}}=0$ (scenario 1), I evaluated the type I error rates given the significance level of 3.6E-10. To test for the validity of this method given a nonnegligible SNP main effect, I deliberately used rs4144003 (on chromosome 16) as SNP2, which had the largest MAF (0.393 in TWB2, and 0.397 in TWB1) among the four SNPs. Power was assessed at $\beta_{G_{1}}=\beta_{G_{2}}=\beta_{\mathrm{INT}}=0.3$ . When evaluating power, the four SNPs were (in turn) regarded as SNP2, constructing scenarios 2–5. A total of 10^8^ replications were conducted under this situation, and each scenario was investigated with $2\times 10^{7}$ simulation replicates.

To sum up, a total of $4\times 10^{8}$ simulation replicates were performed. Each of the following four situations was evaluated with $10^{8}$ replications: TWB2 without SNP main effects; TWB2 with SNP main effects; TWB1 without SNP main effects; and TWB1 with SNP main effects. In each situation, scenario 1 (in the absence of GxG) and scenarios 2–5 (in the presence of GxG) were simulated to assess the type I error rates and power, respectively. Each scenario (under each situation) was investigated with $2\times 10^{7}$ replicates.

### 2.5 GxG analysis for any two vQTL SNPs

For any two vQTL SNPs, RINT-trait was regressed on the numbers of minor alleles (0, 1, or 2) of the two SNPs and their product term (interaction term) while adjusting for the same covariates: sex (male vs. female), age (in years), BMI (in kg/m^2^), performing regular exercise (yes vs. no), educational attainment (an integer from 1 to 7), smoking status (yes vs. no), drinking status (yes vs. no), and the first 10 ancestry PCs. Similarly, when analyzing the five obesity traits (BMI, BFP, WC, HC, and WHR), BMI was excluded from the covariates. To avoid the multicollinearity problem, I calculated the variance inflation factor (VIF) of each regression model using the R package “*car*” (Fox and Weisberg, 2018). A VIF <5 was considered acceptable, which was commonly used as the VIF cutoff value (Rogerson, 2001).

## 3 Results

### 3.1 Simulation results

**FIGURE 1:**
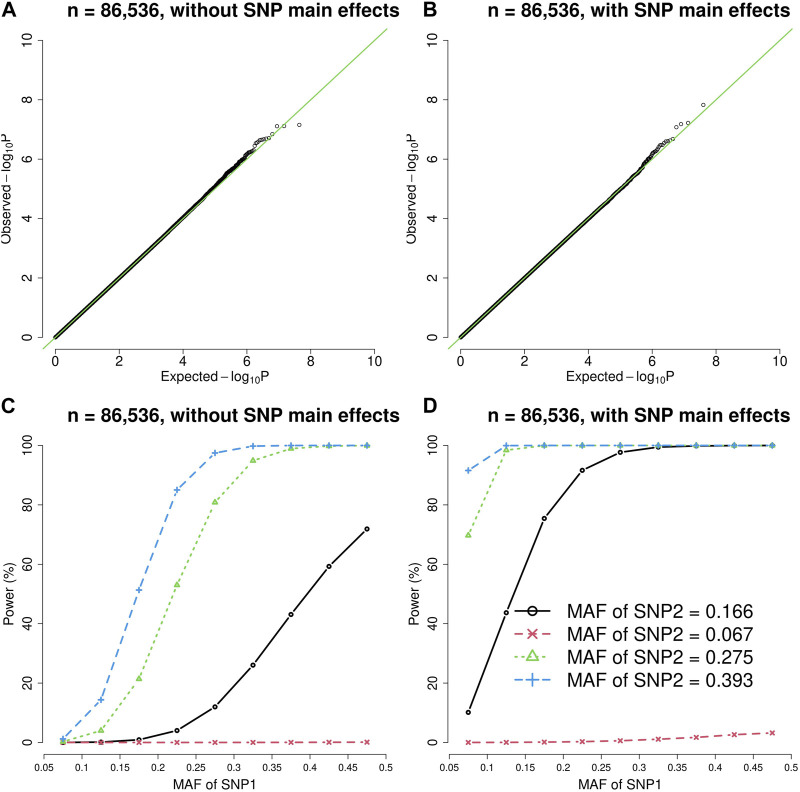
The quantile-quantile (QQ) plots and power curves of the vQTL method when *n* = 86,536. The figures in the top row are QQ plots in the absence of epistasis, without SNP main effects **(A)** and with SNP main effects **(B)**. The bottom row shows the power curves (significance level = 3.6E-10), without SNP main effects **(C)** and with SNP main effects **(D)**. As expected, larger MAF at SNP1 or SNP2 boosted the statistical power.

**FIGURE 2:**
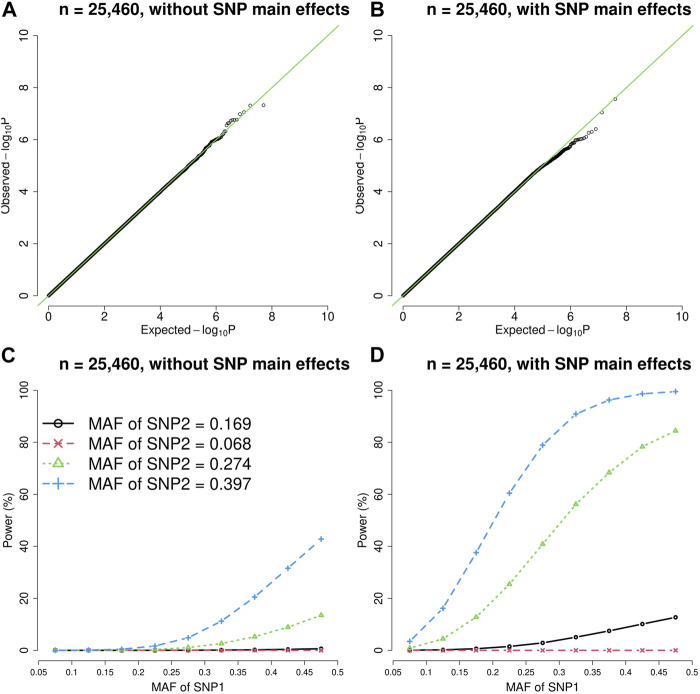
The quantile-quantile (QQ) plots and power curves of the vQTL method when *n* = 25,460. The figures in the top row are QQ plots in the absence of epistasis, without SNP main effects **(A)** and with SNP main effects **(B)**. The bottom row shows the power curves (significance level = 3.6E-10), without SNP main effects **(C)** and with SNP main effects **(D)**. As expected, larger MAF at SNP1 or SNP2 boosted the statistical power.

Figures 1, 2 show the quantile-quantile (QQ) plots and power curves of the vQTL method, where the power was evaluated at α = 3.6E-10. With $4\times 2\times 10^{7}$ simulation replicates for scenario 1 under the four situations (TWB2 without SNP main effects; TWB2 with SNP main effects; TWB1 without SNP main effects; and TWB1 with SNP main effects), no type I errors were observed given α = 3.6E-10. The QQ plots (Figures 1, 2A) showed that the observed *p*-values matched the expected *p*-values under the null hypothesis (no epistasis). The type I error rates were well protected given no *p*-value <3.6E-10.

As shown by Figures 1, 2, the presence of SNP main effects boosted the statistical power (D compared with C) while preserving the validity of the vQTL method (B). As expected, a larger MAF at SNP1 or SNP2 also increased the power. Moreover, the vQTL method is also capable of detecting “pure epistasis” (without individual SNP main effects) (Russ et al., 2022), as demonstrated by Figures 1, 2C.

### 3.2 Scale test to identify vQTLs

I identified 1,668 vQTL SNPs from all 29 TWB2 phenotypes (*p* < 3.6E-10). I then performed the scale test on these 1,668 SNPs using the TWB1 cohort. If the *p*-value <0.05/1,668 = 3.0E-5, the SNP was considered to be successfully replicated in the TWB1. I further used the PLINK clumping command (Purcell et al., 2007) to find 18 nearly independent vQTL SNPs with linkage disequilibrium measure *r* ^*2*^ < 0.01 (Table 1). Only 9 out of the 29 phenotypes demonstrated evidence of vQTLs, including three lipid traits: LDL (3 vQTLs), TCHO (1 vQTL), and TG (1 vQTL); three blood traits: RBC (4 vQTLs), WBC (1 vQTL), and HB (1 vQTL); a kidney trait: UA (1 vQTL); a liver trait: TB (3 vQTLs); and a diabetes trait: FG (3 vQTLs). Except for *APOA5*-rs2075291, all vQTLs were also QTLs (the last column of Table 1).

**TABLE 1:** 18 variance quantitative trait loci (vQTLs).

Phenotype	Chr.	Base pair	SNP	Gene	Major allele/Minor allele	MAF (TWB2/TWB1)	vQTL (scale) test *p*-value (TWB2/TWB1)	QTL (location) test *p*-value (TWB2/TWB1)
LDL	2	21239866	rs150856817	*APOB*	T/A	0.166/0.169	4.8E-12/2.6E-5	5.8E-76/1.4E-13
	11	116661392	rs2075291	*APOA5*	C/A	0.067/0.068	3.1E-16/3.6E-8	0.989/0.015
	19	45416741	rs438811	*APOC1*	C/T	0.175/0.171	5.5E-142/8.1E-41	1.8E-270/3.0E-88
TCHO	19	45400747	rs61679753	*TOMM40*	T/A	0.074/0.071	3.8E-65/5.3E-6	0/4.9E-94
TG	11	116663707	rs662799	*APOA5*	A/G	0.275/0.274	6.6E-26/7.3E-8	0/1.0E-276
RBC	16	268955	rs143660108	*LUC7L*	G/C	0.112/0.114	0/4.4E-144	0/9.2E-140
	16	359953	rs2301522	*AXIN1*	A/G	0.324/0.333	5.4E-45/3.2E-14	1.9E-41/8.1E-12
	16	456841	rs7197553	*DECR2*	T/C	0.468/0.489	1.3E-49/1.7E-10	4.7E-40/1.4E-15
	16	645968	rs4144003	*RAB40C*	T/C	0.393/0.397	2.4E-29/1.6E-8	2.1E-18/5.6E-6
WBC	8	70739986	rs4738028	*SLCO5A1*	T/G	0.457/0.467	5.5E-38/4.0E-7	1.6E-156/7.8E-13
HB	16	279723	rs966965120	*LUC7L*	G/A	0.111/0.113	1.3E-18/3.7E-7	3.1E-116/7.4E-53
UA	4	89046202	rs141471965	*ABCG2*	C/T	0.314/0.318	2.6E-44/2.3E-13	2.5E-257/1.2E-133
TB	2	234346660	rs2242102	*DGKD*	G/A	0.390/0.389	7.7E-14/2.2E-7	7.8E-94/2.5E-34
	2	234671462	rs28946889	*UGT1A10*	G/T	0.420/0.424	1.7E-218/5.3E-57	0/0
	2	234724510	rs28948393	*MROH2A*	T/G	0.157/0.171	6.8E-19/4.3E-9	3.6E-57/8.1E-23
FG	2	169743220	rs143848901	*SPC25*	G/A	0.430/0.442	1.2E-14/1.7E-7	2.1E-67/2.4E-24
	2	169752640	rs76462791	*SPC25*	G/C	0.090/0.088	2.4E-21/9.7E-6	1.9E-54/8.6E-25
	7	44232833	rs741037	*GCK*	G/A	0.198/0.198	6.4E-18/1.9E-6	2.3E-124/9.4E-34

Five vQTLs identified from lipid traits are located in the *APOB*, *APOA5*, *APOC1*, and *TOMM40* genes (Table 1). Many of these vQTL SNPs have been reported to be associated with complex diseases or to exhibit interactions with other SNPs. For example, *APOA5*-rs662799 presented a solid association with TG levels in various ethnicity samples (Liu et al., 2012), and this SNP was identified as a TG-vQTL and a TG-QTL through my analysis (Table 1). Korean data showed that the two vQTL SNPs in the *APOA5* gene, rs2075291 and rs662799 (Table 1), were associated with increased arterial stiffness and decreased adiponectin levels (Kim et al., 2018). 13 Alzheimer’s disease (AD) GWAS cohorts demonstrated that the vQTL SNP in the *APOC1* gene, rs438811, significantly interacted with the *APOE*-ε4 allele. Carrying one minor allele T of rs438811 increased the AD risk by 26.75% in *APOE*-ε4 carriers (Zhang et al., 2018). Data recruited from China’s Second Affiliated Hospital of Xi’an Jiaotong University showed that *AXIN1*-rs2301522 was significantly associated with the risk of osteoporosis (Cui et al., 2022).

### 3.3 Location tests to identify QTLs

While vQTLs were searched through the scale test, QTLs were identified by the location test (testing whether the phenotype mean was dependent on the genotypes) (Staley et al., 2022). Some previous searches for genome-wide epistasis were based on QTLs (Bocianowski, 2013; Laurie et al., 2014; Yang et al., 2018). As a comparison, I also searched for epistasis through the QTL approach as follows. Specifically, I regressed RINT-trait on the number of minor alleles (0, 1, or 2) for each SNP respectively (one SNP at a time) while adjusting for the same 17 covariates, including sex (male vs. female), age (in years), BMI (in kg/m^2^), performing regular exercise (yes vs. no), educational attainment (an integer from 1 to 7), smoking status (yes vs. no), drinking status (yes vs. no), and the first 10 ancestry PCs. The R code for QTL testing can also be downloaded from https://github.com/WanYuLin/Univariate-scale-test-UST.

**FIGURE 3:**
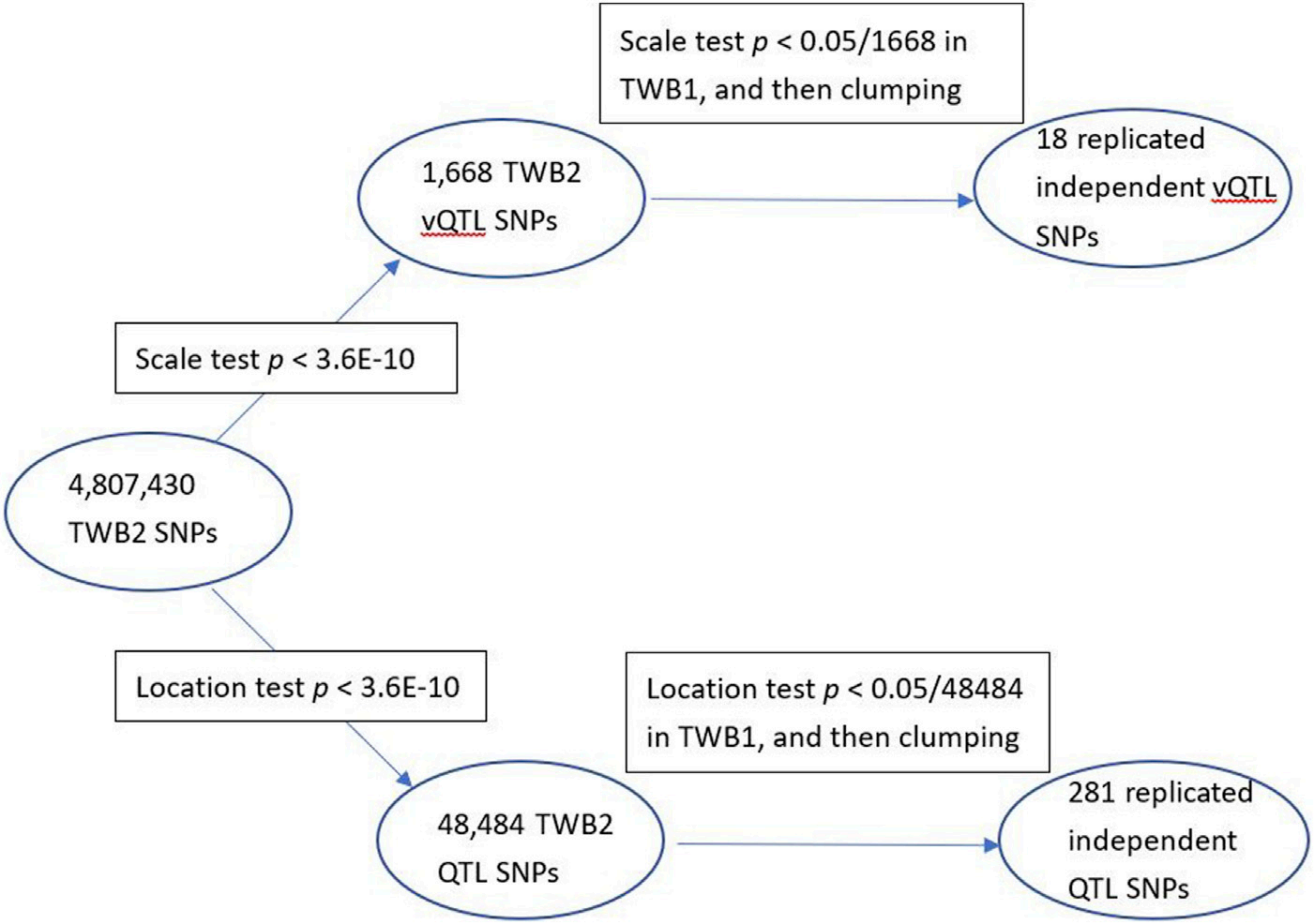
vQTL and QTL approaches to search for epistasis.

I found 48,484 QTL SNPs (*p* < 0.05/(4,807,430 × 29) = 3.6E-10) from the 4,807,430 TWB2 common variants (MAF ≥ 5%). I then performed the location test on these 48,484 SNPs using the TWB1 cohort. If the *p*-value <0.05/48,484 = 1.0E-6, the SNP was successfully replicated in the TWB1. I further used the PLINK clumping command (Purcell et al., 2007) to find 281 nearly independent QTLs with linkage disequilibrium measure *r* ^*2*^ < 0.01. Figure 3 compares the vQTL and QTL approaches to search for epistasis. When resorting to the QTL approach, I also found that the SNPs exhibiting GxG were the vQTL SNPs in Table 1 (or located in the same genes as the vQTL SNPs). Hence, the vQTL approach is more efficient as only $\left(\begin{array}{c} 18\\ 2 \end{array}\right)=153$ GxG tests were performed for each phenotype, compared with $\left(\begin{array}{c} 281\\ 2 \end{array}\right)=39340$ SNP pairs analyzed for the QTL method.

### 3.4 Results of GxG analysis for any two vQTL SNPs

According to the 18 vQTLs listed in Table 1, $(\begin{array}{c} 18\\ 2 \end{array})=153$ GxG tests were performed for each phenotype, and 4,437 (=153 × 29) GxG tests were implemented for 29 phenotypes. Among the 4,437 tests, 15 were significant at *p* < 0.05/4437 = 1.1E-5 in the discovery (TWB2) cohort (Table 2). These 15 GxG interactions were further analyzed using the replication (TWB1) cohort, and the GxG interaction *p*-values were all less than 0.02 (Table 2). When resorting to the false discovery rate control (FDR) procedure (Benjamini and Hochberg, 1995), the FDRs were all less than 2%. Controlling FDR at 5% is commonly adopted in genomic studies (Sun et al., 2006). Therefore, an FDR level of 2% was acceptable, and all 15 GxG were replicated using the TWB1 cohort. Furthermore, the directions of GxG interaction coefficients (synergistic or antagonistic interaction effects) were consistent across the two cohorts. Because the 18 vQTL SNPs were nearly independent with linkage disequilibrium measure *r* ^*2*^ < 0.01, putting any two and their interaction (product) term into the model did not cause multicollinearity problems. The VIFs of all regression models were controlled under 2 (Table 2).

**TABLE 2:** 15 GxG interactions identified from 6 phenotypes.

Phenotype	SNP x SNP	G x G	*r* ^2^ between the two SNPs (TWB2/TWB1)	*G* x *G interaction p*-value^a^ (TWB2/TWB1)	*GxG interaction* coefficient (TWB2/TWB1)	Variance inflation factor (VIF) (TWB2/TWB1)
TCHO	rs662799-rs61679753	*APOA5*-*TOMM40*	7.1E-7/2.1E–6	3.3E-15/4.1E-3	0.11/0.08	1.32/1.34
FG	rs143848901-rs76462791	*SPC25-SPC25*	2.3E-4/1.7E–4	7.1E-25/5.2E-9	0.11/0.11	1.32/1.34
TB	rs2242102-rs28946889	*DGKD-UGT1A10*	2.7E-3/2.8E–3	2.9E-25/2.6E-12	−0.06/−0.08	1.32/1.34
	rs28946889-rs28948393	*UGT1A10-MROH2A*	5.6E-3/8.1E-3	7.5E-17/5.5E-7	0.07/0.07	1.32/1.34
RBC	rs143660108-rs2301522	*LUC7L-AXIN1*	2.8E-4/1.4E–4	4.4E-84/2.2E-26	−0.19/−0.18	1.32/1.34
	rs143660108-rs7197553	*LUC7L-DECR2*	2.5E-9/1.1E–5	2.0E-150/1.9E-54	0.24/0.25	1.32/1.34
	rs143660108-rs4144003	*LUC7L-RAB40C*	6.7E-4/5.4E–4	7.9E-45/2.0E-14	0.13/0.12	1.32/1.34
	rs2301522-rs7197553	*AXIN1-DECR2*	1.4E-3/2.9E–3	5.6E-6/2.9E-4	−0.03/−0.04	1.32/1.34
	rs2301522-rs4144003	*AXIN1-RAB40C*	3.7E-3/3.1E–3	2.6E-6/3.5E-3	−0.03/−0.03	1.32/1.34
	rs7197553-rs4144003	*DECR2-RAB40C*	4.7E-3/2.0E–3	1.1E-7/2.8E-3	0.03/0.03	1.32/1.34
HB	rs143660108-rs2301522	*LUC7L-AXIN1*	2.8E-4/1.4E–4	2.0E-23/2.6E-16	0.09/0.12	1.32/1.34
	rs143660108-rs7197553	*LUC7L-DECR2*	2.5E-9/1.1E–5	4.3E-42/8.1E-8	−0.11/−0.08	1.32/1.34
	rs143660108-rs4144003	*LUC7L-RAB40C*	6.7E-4/5.4E–4	6.3E-11/6.4E-4	−0.05/−0.05	1.32/1.34
HCT	rs143660108-rs2301522	*LUC7L-AXIN1*	2.8E-4/1.4E–4	2.5E-6/1.3E-6	0.04/0.07	1.32/1.34
	rs143660108-rs7197553	*LUC7L-DECR2*	2.5E-9/1.1E–5	1.0E-14/0.020	−0.07/−0.04	1.32/1.34

^a^ Because 18 vQTLs were detected, I performed $\left(\begin{array}{c} 18\\ 2 \end{array}\right)\times 29=4437$ GxG tests for any two vQTLs. In the discovery cohort (TWB2), GxG interactions were considered significant with *p*-values <0.05/4437 = 1.1E-5. The false discovery rates were all <2% in the replication cohort (TWB1).

**FIGURE 4:**
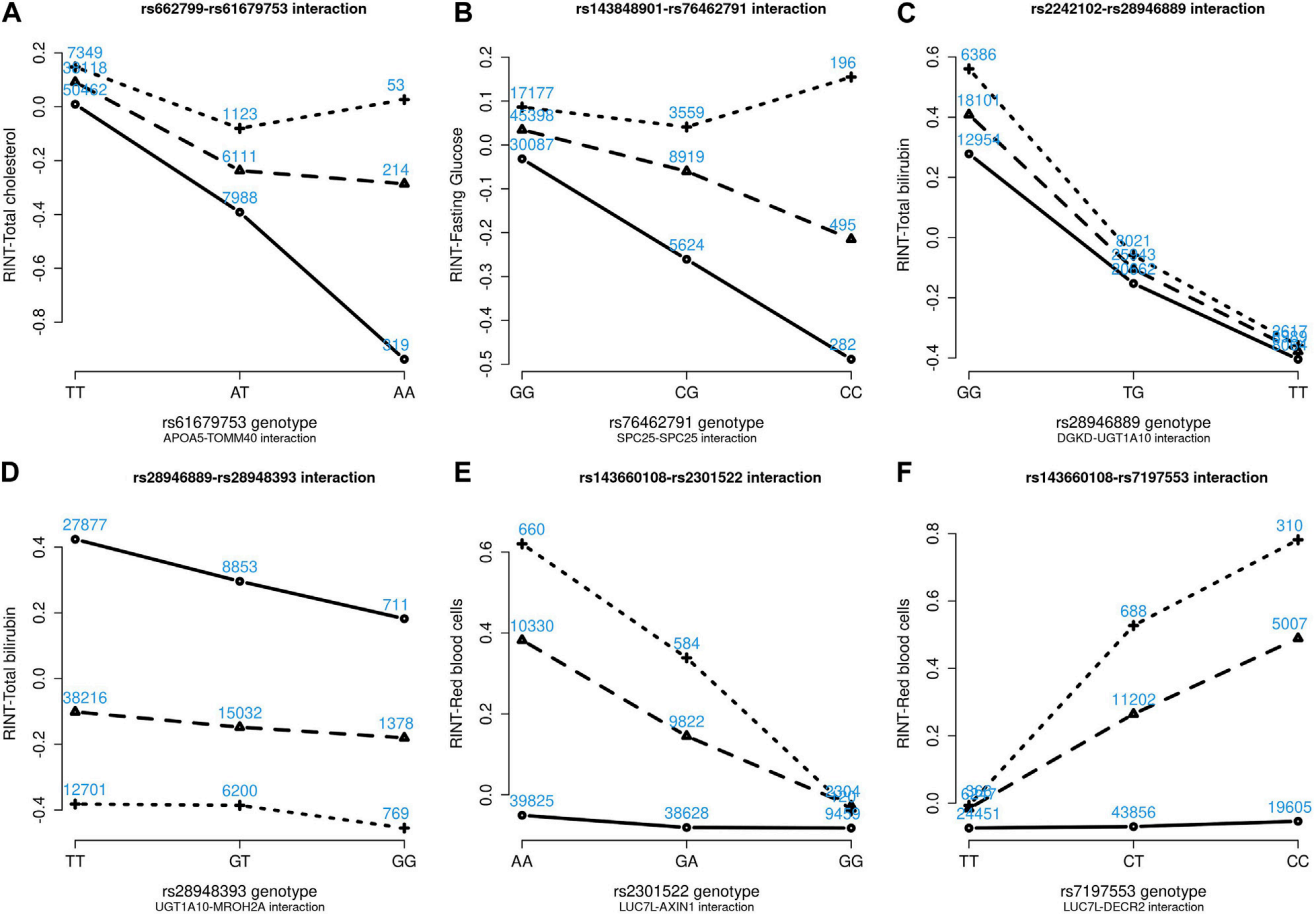
Six GxG interaction plots for total cholesterol **(A)**, fasting glucose **(B)**, total bilirubin **(C, D)**, and red blood cells **(E, F)**. **(A)** Represents rs662799-rs61679753 interaction plot combining the TWB2 and TWB1 cohorts, where the *x*-axis denotes the three genotypes of rs61679753, and the *y*-axis calibrates the mean RINT-total cholesterol. The solid, dashed, and dotted lines mark the three genotypes of rs662799: AA (two major alleles), AG, and GG (two minor alleles), respectively. The blue number shown around each point is the sample size of that genotype combination. Other plots were made similarly.

**FIGURE 5:**
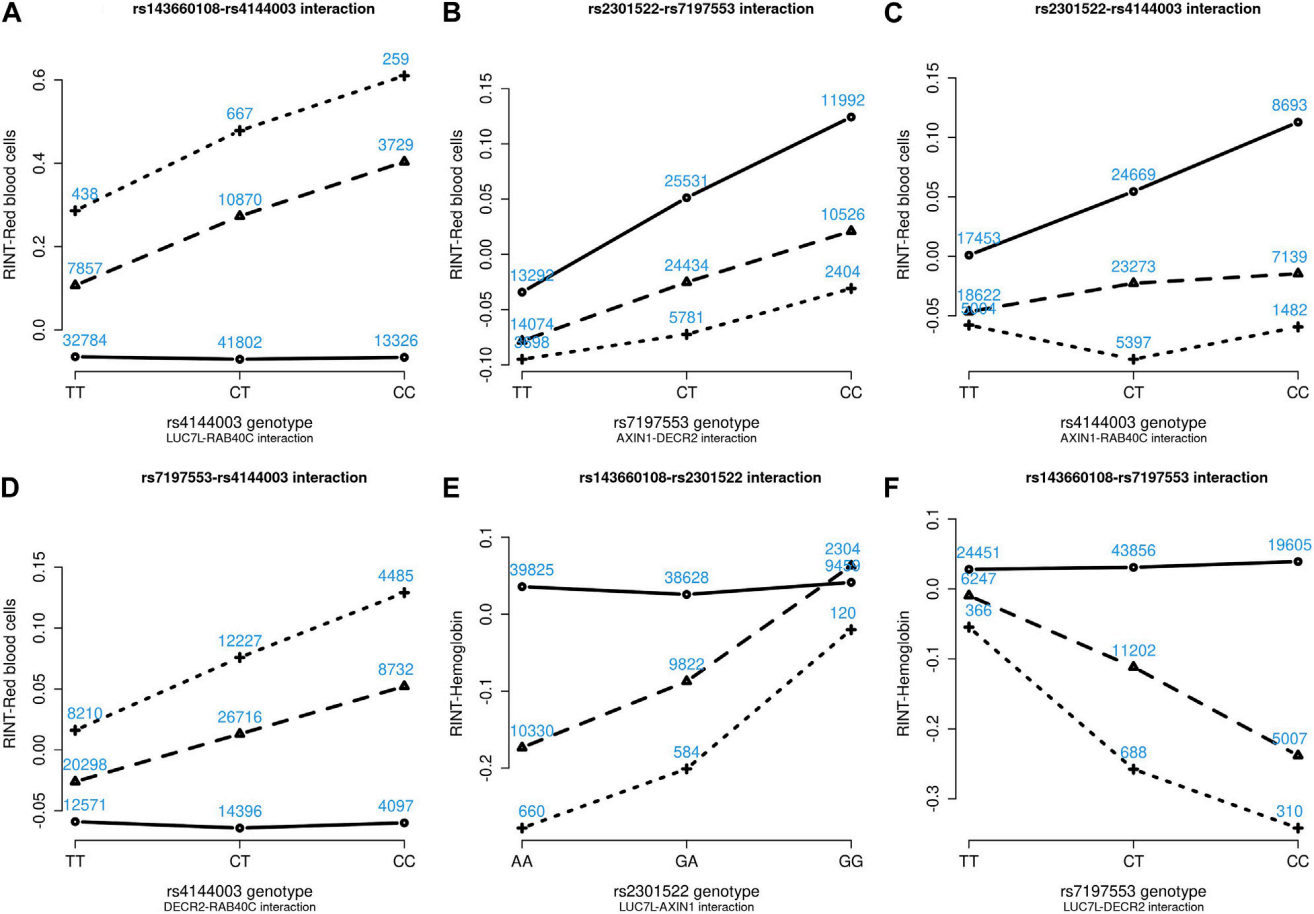
Six GxG interaction plots for red blood cells **(A–D)** and hemoglobin **(E, F)**. **(A)** Represents rs143660108-rs4144003 interaction plot combining the TWB2 and TWB1 cohorts, where the *x*-axis denotes the three genotypes of rs4144003, and the *y*-axis calibrates the mean RINT-red blood cells. The solid, dashed, and dotted lines mark the three genotypes of rs143660108: GG (two major alleles), CG, and CC (two minor alleles), respectively. The blue number shown around each point is the sample size of that genotype combination. Other plots were made similarly.

**FIGURE 6:**
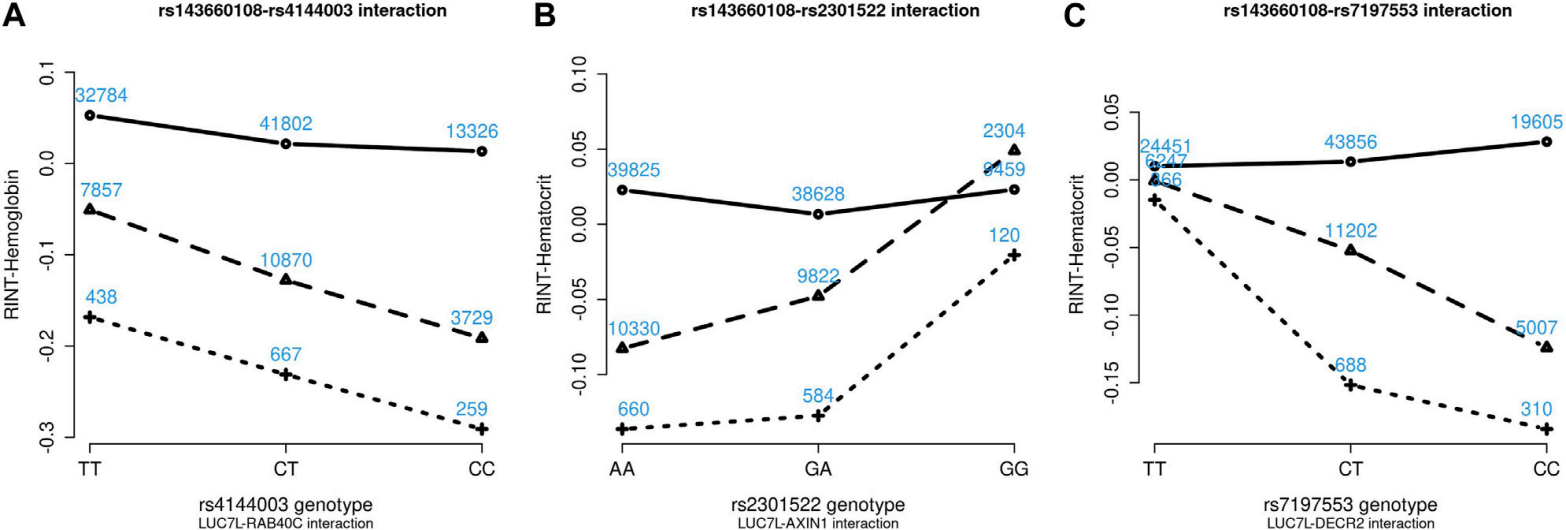
Three GxG interaction plots for hemoglobin **(A)** and hematocrit **(B, C)**. **(A)** Represents rs143660108-rs4144003 interaction plot combining the TWB2 and TWB1 cohorts, where the *x*-axis denotes the three genotypes of rs4144003, and the *y*-axis calibrates the mean RINT-hemoglobin. The solid, dashed, and dotted lines mark the three genotypes of rs143660108: GG (two major alleles), CG, and CC (two minor alleles), respectively. The blue number shown around each point is the sample size of that genotype combination. Other plots were made similarly.

Figures 4–6 show the GxG interaction plots for TCHO (1 GxG), FG (1 GxG), TB (2 GxG), RBC (6 GxG), HB (3 GxG), and HCT (2 GxG). The *y*-axis represents the averages of RINT-trait of nine genotype combinations of two SNPs. Lines with different slopes suggest that the effect of an SNP depends on the genotype of another SNP, which is a clue of GxG. Nonetheless, these interaction plots may not completely correspond to the GxG interaction *p*-values (Table 2). Unlike the epistasis test results (Table 2), these plots are descriptive summaries without adjusting for any covariate. For example, lines converging at genotype GG of rs2301522 (Figure 4E) represented that individuals with rs2301522-GG had similar RBC, while individuals with rs2301522-AA (or rs2301522-AG) had divergent RBC depending on rs143660108’s genotypes. Lines showing crosscut (Figure 5E) indicated “cross-over interaction,” meaning that rs143660108’s genotypes with the larger mean HB switched over at rs2301522-GG.

All GxG were observed for gene pairs on the same chromosome, except for the *APOA5* (chromosome 11)—*TOMM40* (chromosome 19) interaction for TCHO. Both *APOA5* and *TOMM40* are involved in lipid metabolism (Hishida et al., 2014). The GxG analysis showed that minor alleles of *APOA5*-rs662799 and *TOMM40*-rs61679753 exhibited significant synergistic interaction on TCHO (Figure 4A).

Because all phenotypes were RINT-transformed before the analysis, the 15 GxG interaction coefficients (Table 2) could be directly compared. Both cohorts suggested that *LUC7L*-rs143660108 and *DECR2*-rs7197553 presented the most substantial interaction on RBC. The minor alleles of these two SNPs exhibited a notable synergistic interaction on RBC (Figure 4F).

### 3.5 Reactome pathway analysis results

Table 3 shows the Reactome pathway analysis results on genes identified within the same trait category. Many lipid-related pathways were enriched (FDR <0.05) in the vQTLs identified from lipid traits (*APOB*, *APOA5*, *APOC1*, and *TOMM40*). “Glucuronidation” and “Paracetamol ADME” (Absorption, Distribution, Metabolism, Excretion) pathways were over-represented in the vQTLs of liver traits (*DGKD*, *UGT1A10*, and *MROH2A*). Glucuronidation is a major metabolic reaction in the liver (Yang et al., 2017), while paracetamol is also extensively metabolized in this organ (Forrest et al., 1982). Pathways related to beta cells were enriched in diabetes’ vQTLs (*SPC25* and *GCK*). Beta cells are critical to diabetes by producing insulin to control blood glucose levels (Cerf, 2013). These pathway analysis results showed that the vQTLs found in this study were highly relevant to the traits.

**TABLE 3:** Reactome pathway analysis results on genes identified within the same trait category.

Trait category	vQTLs	Pathway name^a^	FDR^b^
Lipid traits	*APOB*	Plasma lipoprotein assembly, remodeling, and clearance	1.3E-4
	*APOA5*	VLDL clearance	2.4E-4
	*APOC1*	Plasma lipoprotein remodeling	5.1E-4
	*TOMM40*	VLDL assembly	2.7E-3
Blood traits	*LUC7L*, *AXIN1*, *DECR2*, *RAB40C*, *SLCO5A1*	Deletions in the AXIN1 gene destabilize the destruction complex	0.062
		RUNX1 regulates transcription of genes involved in WNT signaling	0.062
Liver traits	*DGKD*	Glucuronidation [Glucuronidation is a major metabolic reaction that mainly occurs in the liver (Yang et al., 2017)]	0.02
	*UGT1A10*	Effects of PIP2 hydrolysis	0.02
	*MROH2A*	Paracetamol ADME (Absorption, Distribution, Metabolism, Excretion) [Paracetamol is extensively metabolized in the liver (Forrest et al., 1982)]	0.02
Diabetes traits	*SPC25*	Defective GCK causes maturity-onset diabetes of the young 2 (MODY2)	0.04
	*GCK*	Regulation of gene expression in beta cells (Beta cells produce insulin in response to blood glucose levels)	0.04
		Regulation of beta-cell development	0.04
		FOXO-mediated transcription of oxidative stress, metabolic and neuronal genes [FOXO proteins are essential to maintain the differentiation of beta cells (Marchelek-Mysliwiec et al., 2022)]	0.04

^a^ The pathway names presented in the bold type were the names from Reactome. Some pathways were further explained in detail (unbold type).

^b^ FDR: Benjamini–Hochberg false discovery rate (Benjamini and Hochberg, 1995). Many lipid-related pathways were enriched (FDR <0.05) in the vQTLs identified from lipid traits. The four leading pathways were listed to save space. For other trait categories, the leading pathways having FDR with ties were presented.

## 4 Discussion

In this work, 11 GxG were detected for blood traits including RBC, HB, and HCT; 2 for TB (liver trait); 1 for FG (diabetes trait); and 1 for TCHO (lipid trait). Among the 15 significant GxG, 8 demonstrated synergistic interaction effects, while the other 7 GxG exhibited antagonistic interaction effects. The interaction directions for 15 GxG were consistent across the two TWB cohorts (Table 2).

A computationally feasible GxG approach will facilitate the discovery of critical epistasis. This study provided a viable way to search for epistasis genome-wide, and I have applied this approach to 29 phenotypes. With this vQTL method, SNPs presenting epistasis will not be overlooked because of the lack of marginal effects.

The vQTL method can identify interactions with marginal effects (Figures 1, 2D) and can also detect pure epistasis (Figures 1, 2C). As derived by Equation 2, $Var\left(\left.Y\right|G_{1}=g_{1}\right)={\left(\beta_{G_{2}}+\beta_{\mathrm{INT}}g_{1}\right)}^{2}Var\left(G_{2}\right)+Var\left(\varepsilon \right)$ . Nonetheless, a non-zero SNP2’s main effect $\beta_{G_{2}}$ can enlarge the difference in trait variation across the three genotypes of SNP1. That is why the power in Figures 1, 2D is higher than in Figures 1, 2C.

Although the associations between diseases and low-frequency or rare variants have been investigated over the past decade (Bomba et al., 2017), I here only analyzed SNPs with MAFs ≥ 5%. The main reason is that I aim to provide more solid evidence of epistasis that can be replicated in an independent cohort (here, TWB1). GxG is a topic that explores the impacts of joint distribution of SNP pairs on phenotypes. If the sample size of any genotype combination (from an SNP pair) is too small, this GxG signal is unreliable and can hardly be replicated in another cohort. Therefore, GxG or GxE studies usually focus on more common SNPs. For example, a systematic GxE search through vQTLs of 13 continuous traits from the UK Biobank also focused on SNPs with MAFs ≥ 5% (Wang et al., 2019), the same MAF cutoff as this study. A robust GxG analysis method for low-frequency or rare variants still requires further research.

## Data Availability

The individual-level Taiwan Biobank data supporting the findings in this study are available upon application to Taiwan Biobank (https://www.twbiobank.org.tw/new_web/). Taiwan Biobank approved my application to access the data on February 18, 2020 (application number: TWBR10810-07; principal investigator: W-YL).
